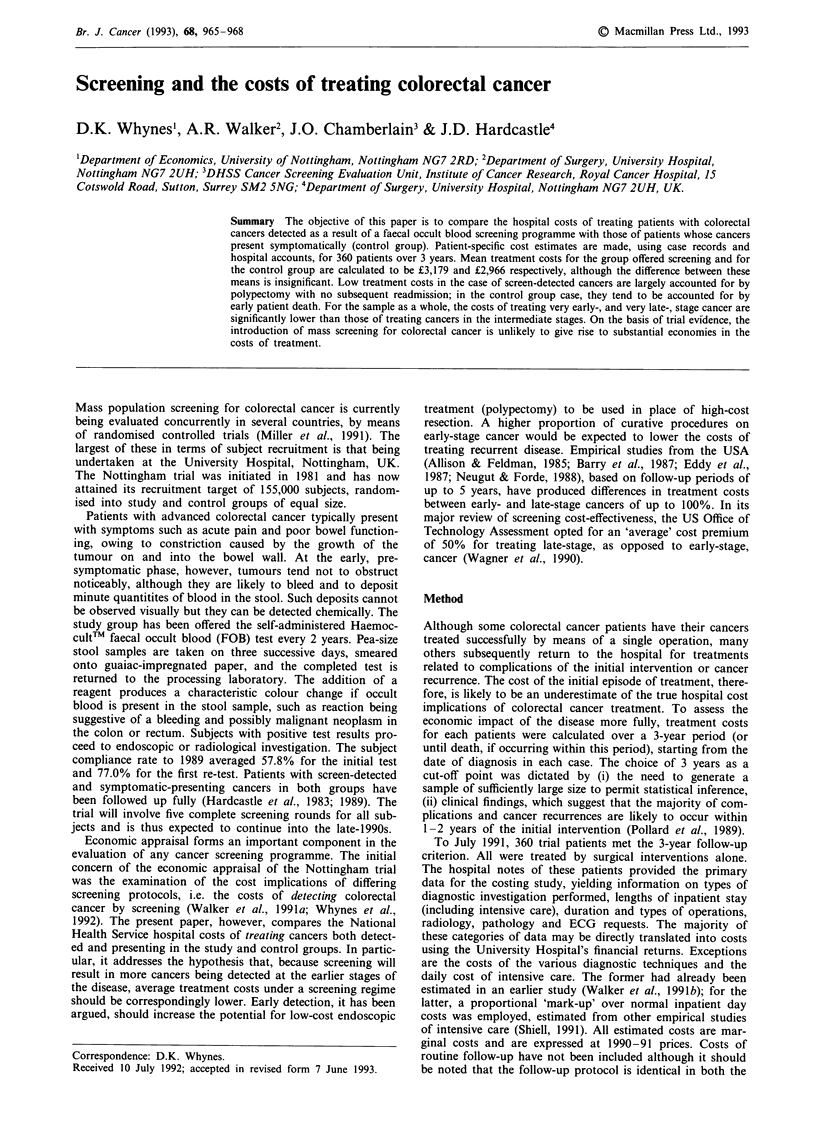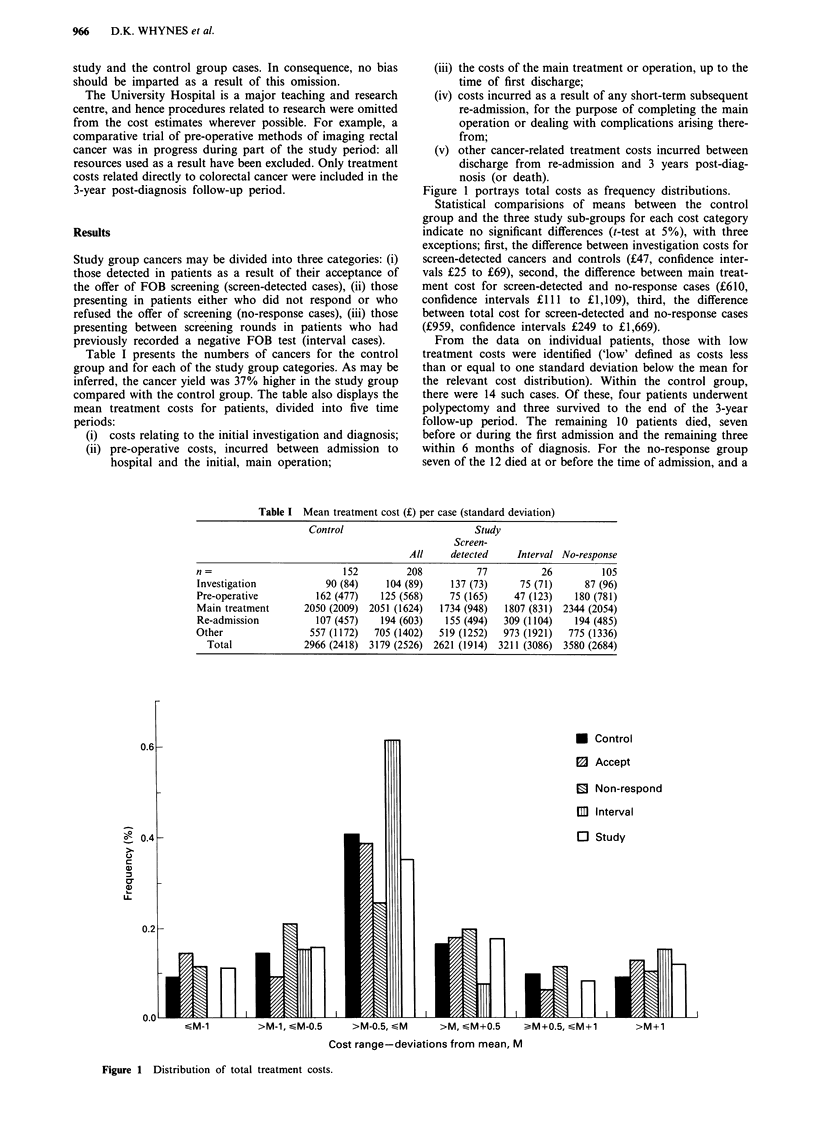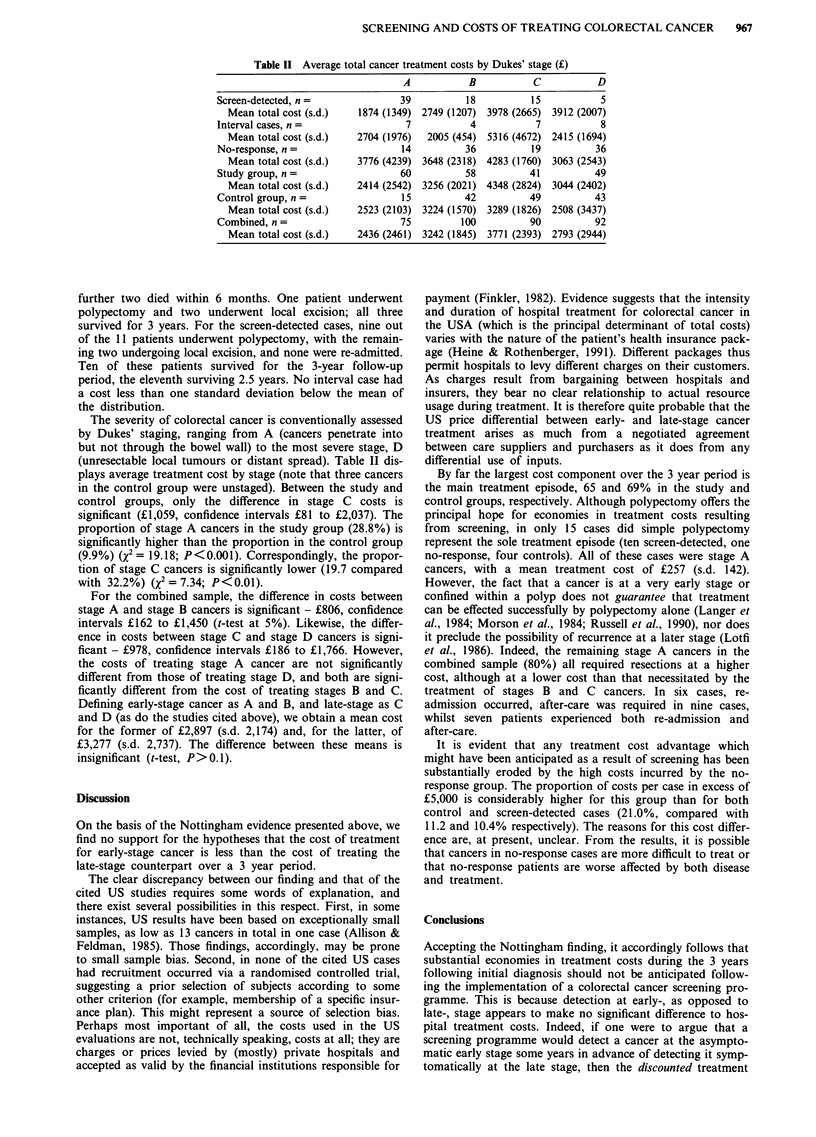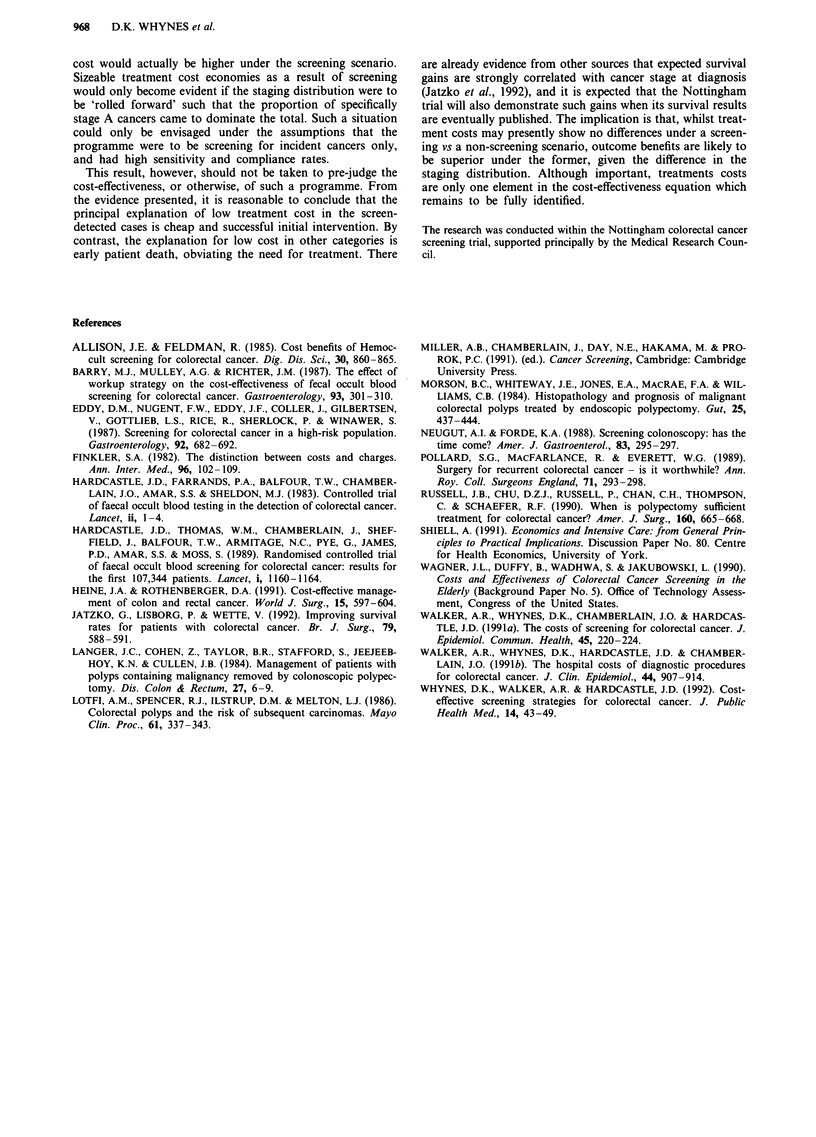# Screening and the costs of treating colorectal cancer.

**DOI:** 10.1038/bjc.1993.462

**Published:** 1993-11

**Authors:** D. K. Whynes, A. R. Walker, J. O. Chamberlain, J. D. Hardcastle

**Affiliations:** Department of Economics, University of Nottingham, UK.

## Abstract

The objective of this paper is to compare the hospital costs of treating patients with colorectal cancers detected as a result of a faecal occult blood screening programme with those of patients whose cancers present symptomatically (control group). Patient-specific cost estimates are made, using case records and hospital accounts, for 360 patients over 3 years. Mean treatment costs for the group offered screening and for the control group are calculated to be 3,179 pounds and 2,966 pounds respectively, although the difference between these means is insignificant. Low treatment costs in the case of screen-detected cancers are largely accounted for by polypectomy with no subsequent readmission; in the control group case, they tend to be accounted for by early patient death. For the sample as a whole, the costs of treating very early-, and very late-, stage cancer are significantly lower than those of treating cancers in the intermediate stages. On the basis of trial evidence, the introduction of mass screening for colorectal cancer is unlikely to give rise to substantial economies in the costs of treatment.


*Br. J. Cancer* (1993), **68**, 965–968 © Macmillan Press Ltd., 1993

### Screening and the costs of treating colorectal cancer

D.K. Whynes[1], A.R. Walker[2], J.O. Chamberlain[3] & J.D. Hardcastle[4]

[1]*Department of Economics, University of Nottingham, Nottingham NG7 2RD;* [2]*Department of Surgery, University Hospital, Nottingham NG7 2UH;* [3]*DHSS Cancer Screening Evaluation Unit, Institute of Cancer Research, Royal Cancer Hospital, 15 Cotswold Road, Sutton, Surrey SM2 5NG;* [4]*Department of Surgery, University Hospital, Nottingham NG7 2UH, UK.*

**Summary** The objective of this paper is to compare the hospital costs of treating patients with colorectal cancers detected as a result of a faecal occult blood screening programme with those of patients whose cancers present symptomatically (control group). Patient-specific cost estimates are made, using case records and hospital accounts, for 360 patients over 3 years. Mean treatment costs for the group offered screening and for the control group are calculated to be £3,179 and £2,966 respectively, although the difference between these means is insignificant. Low treatment costs in the case of screen-detected cancers are largely accounted for by polypectomy with no subsequent readmission; in the control group case, they tend to be accounted for by early patient death. For the sample as a whole, the costs of treating very early-, and very late-, stage cancer are significantly lower than those of treating cancers in the intermediate stages. On the basis of trial evidence, the introduction of mass screening for colorectal cancer is unlikely to give rise to substantial economies in the costs of treatment.

Mass population screening for colorectal cancer is currently being evaluated concurrently in several countries, by means of randomised controlled trials (Miller *et al.*, 1991). The largest of these in terms of subject recruitment is that being undertaken at the University Hospital, Nottingham, UK. The Nottingham trial was initiated in 1981 and has now attained its recruitment target of 155,000 subjects, randomised into study and control groups of equal size.

Patients with advanced colorectal cancer typically present with symptoms such as acute pain and poor bowel functioning, owing to constriction caused by the growth of the tumour on and into the bowel wall. At the early, pre-symptomatic phase, however, tumours tend not to obstruct noticeably, although they are likely to bleed and to deposit minute quantitites of blood in the stool. Such deposits cannot be observed visually but they can be detected chemically. The study group has been offered the self-administered Haemoccult™ faecal occult blood (FOB) test every 2 years. Pea-size stool samples are taken on three successive days, smeared onto guaiac-impregnated paper, and the completed test is returned to the processing laboratory. The addition of a reagent produces a characteristic colour change if occult blood is present in the stool sample, such as reaction being suggestive of a bleeding and possibly malignant neoplasm in the colon or rectum. Subjects with positive test results proceed to endoscopic or radiological investigation. The subject compliance rate to 1989 averaged 57.8% for the initial test and 77.0% for the first re-test. Patients with screen-detected and symptomatic-presenting cancers in both groups have been followed up fully (Hardcastle *et al.*, 1983; 1989). The trial will involve five complete screening rounds for all subjects and is thus expected to continue into the late-1990s.

Economic appraisal forms an important component in the evaluation of any cancer screening programme. The initial concern of the economic appraisal of the Nottingham trial was the examination of the cost implications of differing screening protocols, i.e. the costs of *detecting* colorectal cancer by screening (Walker *et al.*, 1991*a*; Whynes *et al.*, 1992). The present paper, however, compares the National Health Service hospital costs of *treating* cancers both detected and presenting in the study and control groups. In particular, it addresses the hypothesis that, because screening will result in more cancers being detected at the earlier stages of the disease, average treatment costs under a screening regime should be correspondingly lower. Early detection, it has been argued, should increase the potential for low-cost endoscopic treatment (polypectomy) to be used in place of high-cost resection. A higher proportion of curative procedures on early-stage cancer would be expected to lower the costs of treating recurrent disease. Empirical studies from the USA (Allison & Feldman, 1985; Barry *et al.*, 1987; Eddy *et al.*, 1987; Neugut & Forde, 1988), based on follow-up periods of up to 5 years, have produced differences in treatment costs between early- and late-stage cancers of up to 100%. In its major review of screening cost-effectiveness, the US Office of Technology Assessment opted for an 'average' cost premium of 50% for treating late-stage, as opposed to early-stage, cancer (Wagner *et al.*, 1990).

### Method

Although some colorectal cancer patients have their cancers treated successfully by means of a single operation, many others subsequently return to the hospital for treatments related to complications of the initial intervention or cancer recurrence. The cost of the initial episode of treatment, therefore, is likely to be an underestimate of the true hospital cost implications of colorectal cancer treatment. To assess the economic impact of the disease more fully, treatment costs for each patients were calculated over a 3-year period (or until death, if occurring within this period), starting from the date of diagnosis in each case. The choice of 3 years as a cut-off point was dictated by (i) the need to generate a sample of sufficiently large size to permit statistical inference, (ii) clinical findings, which suggest that the majority of complications and cancer recurrences are likely to occur within 1–2 years of the initial intervention (Pollard *et al.*, 1989).

To July 1991, 360 trial patients met the 3-year follow-up criterion. All were treated by surgical interventions alone. The hospital notes of these patients provided the primary data for the costing study, yielding information on types of diagnostic investigation performed, lengths of inpatient stay (including intensive care), duration and types of operations, radiology, pathology and ECG requests. The majority of these categories of data may be directly translated into costs using the University Hospital's financial returns. Exceptions are the costs of the various diagnostic techniques and the daily cost of intensive care. The former had already been estimated in an earlier study (Walker *et al.*, 1991*b*); for the latter, a proportional 'mark-up' over normal inpatient day costs was employed, estimated from other empirical studies of intensive care (Shiell, 1991). All estimated costs are marginal costs and are expressed at 1990–91 prices. Costs of routine follow-up have not been included although it should be noted that the follow-up protocol is identical in both the

Correspondence: D.K. Whynes.
Received 10 July 1992; accepted in revised form 7 June 1993.

study and the control group cases. In consequence, no bias should be imparted as a result of this omission.

The University Hospital is a major teaching and research centre, and hence procedures related to research were omitted from the cost estimates wherever possible. For example, a comparative trial of pre-operative methods of imaging rectal cancer was in progress during part of the study period: all resources used as a result have been excluded. Only treatment costs related directly to colorectal cancer were included in the 3-year post-diagnosis follow-up period.

### Results

Study group cancers may be divided into three categories: (i) those detected in patients as a result of their acceptance of the offer of FOB screening (screen-detected cases), (ii) those presenting in patients either who did not respond or who refused the offer of screening (no-response cases), (iii) those presenting between screening rounds in patients who had previously recorded a negative FOB test (interval cases).

Table I presents the numbers of cancers for the control group and for each of the study group categories. As may be inferred, the cancer yield was 37% higher in the study group compared with the control group. The table also displays the mean treatment costs for patients, divided into five time periods:

(i) costs relating to the initial investigation and diagnosis;
(ii) pre-operative costs, incurred between admission to hospital and the initial, main operation;
(iii) the costs of the main treatment or operation, up to the time of first discharge;
(iv) costs incurred as a result of any short-term subsequent re-admission, for the purpose of completing the main operation or dealing with complications arising therefrom;
(v) other cancer-related treatment costs incurred between discharge from re-admission and 3 years post-diagnosis (or death).

Figure 1 portrays total costs as frequency distributions.

Statistical comparisions of means between the control group and the three study sub-groups for each cost category indicate no significant differences ($t$-test at 5%), with three exceptions; first, the difference between investigation costs for screen-detected cancers and controls (£47, confidence intervals £25 to £69), second, the difference between main treatment cost for screen-detected and no-response cases (£610, confidence intervals £111 to £1,109), third, the difference between total cost for screen-detected and no-response cases (£959, confidence intervals £249 to £1,669).

From the data on individual patients, those with low treatment costs were identified ('low' defined as costs less than or equal to one standard deviation below the mean for the relevant cost distribution). Within the control group, there were 14 such cases. Of these, four patients underwent polypectomy and three survived to the end of the 3-year follow-up period. The remaining 10 patients died, seven before or during the first admission and the remaining three within 6 months of diagnosis. For the no-response group seven of the 12 died at or before the time of admission, and a

**Table I** Mean treatment cost (£) per case (standard deviation)

| | *Control* | *Study* | | | |
|---|---|---|---|---|---|
| | | *All* | *Screen-detected* | *Interval* | *No-response* |
| $n =$ | 152 | 208 | 77 | 26 | 105 |
| Investigation | 90 (84) | 104 (89) | 137 (73) | 75 (71) | 87 (96) |
| Pre-operative | 162 (477) | 125 (568) | 75 (165) | 47 (123) | 180 (781) |
| Main treatment | 2050 (2009) | 2051 (1624) | 1734 (948) | 1807 (831) | 2344 (2054) |
| Re-admission | 107 (457) | 194 (603) | 155 (494) | 309 (1104) | 194 (485) |
| Other | 557 (1172) | 705 (1402) | 519 (1252) | 973 (1921) | 775 (1336) |
| Total | 2966 (2418) | 3179 (2526) | 2621 (1914) | 3211 (3086) | 3580 (2684) |

**Figure 1** Distribution of total treatment costs.

**Table II** Average total cancer treatment costs by Dukes' stage (£)

| | *A* | *B* | *C* | *D* |
|---|---|---|---|---|
| Screen-detected, *n* = | 39 | 18 | 15 | 5 |
| Mean total cost (s.d.) | 1874 (1349) | 2749 (1207) | 3978 (2665) | 3912 (2007) |
| Interval cases, *n* = | 7 | 4 | 7 | 8 |
| Mean total cost (s.d.) | 2704 (1976) | 2005 (454) | 5316 (4672) | 2415 (1694) |
| No-response, *n* = | 14 | 36 | 19 | 36 |
| Mean total cost (s.d.) | 3776 (4239) | 3648 (2318) | 4283 (1760) | 3063 (2543) |
| Study group, *n* = | 60 | 58 | 41 | 49 |
| Mean total cost (s.d.) | 2414 (2542) | 3256 (2021) | 4348 (2824) | 3044 (2402) |
| Control group, *n* = | 15 | 42 | 49 | 43 |
| Mean total cost (s.d.) | 2523 (2103) | 3224 (1570) | 3289 (1826) | 2508 (3437) |
| Combined, *n* = | 75 | 100 | 90 | 92 |
| Mean total cost (s.d.) | 2436 (2461) | 3242 (1845) | 3771 (2393) | 2793 (2944) |

further two died within 6 months. One patient underwent polypectomy and two underwent local excision; all three survived for 3 years. For the screen-detected cases, nine out of the 11 patients underwent polypectomy, with the remaining two undergoing local excision, and none were re-admitted. Ten of these patients survived for the 3-year follow-up period, the eleventh surviving 2.5 years. No interval case had a cost less than one standard deviation below the mean of the distribution.

The severity of colorectal cancer is conventionally assessed by Dukes' staging, ranging from A (cancers penetrate into but not through the bowel wall) to the most severe stage, D (unresectable local tumours or distant spread). Table II displays average treatment cost by stage (note that three cancers in the control group were unstaged). Between the study and control groups, only the difference in stage C costs is significant (£1,059, confidence intervals £81 to £2,037). The proportion of stage A cancers in the study group (28.8%) is significantly higher than the proportion in the control group (9.9%) ($\chi^2 = 19.18$; $P < 0.001$). Correspondingly, the proportion of stage C cancers is significantly lower (19.7 compared with 32.2%) ($\chi^2 = 7.34$; $P < 0.01$).

For the combined sample, the difference in costs between stage A and stage B cancers is significant – £806, confidence intervals £162 to £1,450 (*t*-test at 5%). Likewise, the difference in costs between stage C and stage D cancers is significant – £978, confidence intervals £186 to £1,766. However, the costs of treating stage A cancer are not significantly different from those of treating stage D, and both are significantly different from the cost of treating stages B and C. Defining early-stage cancer as A and B, and late-stage as C and D (as do the studies cited above), we obtain a mean cost for the former of £2,897 (s.d. 2,174) and, for the latter, of £3,277 (s.d. 2,737). The difference between these means is insignificant (*t*-test, $P > 0.1$).

### Discussion

On the basis of the Nottingham evidence presented above, we find no support for the hypotheses that the cost of treatment for early-stage cancer is less than the cost of treating the late-stage counterpart over a 3 year period.

The clear discrepancy between our finding and that of the cited US studies requires some words of explanation, and there exist several possibilities in this respect. First, in some instances, US results have been based on exceptionally small samples, as low as 13 cancers in total in one case (Allison & Feldman, 1985). Those findings, accordingly, may be prone to small sample bias. Second, in none of the cited US cases had recruitment occurred via a randomised controlled trial, suggesting a prior selection of subjects according to some other criterion (for example, membership of a specific insurance plan). This might represent a source of selection bias. Perhaps most important of all, the costs used in the US evaluations are not, technically speaking, costs at all; they are charges or prices levied by (mostly) private hospitals and accepted as valid by the financial institutions responsible for payment (Finkler, 1982). Evidence suggests that the intensity and duration of hospital treatment for colorectal cancer in the USA (which is the principal determinant of total costs) varies with the nature of the patient's health insurance package (Heine & Rothenberger, 1991). Different packages thus permit hospitals to levy different charges on their customers. As charges result from bargaining between hospitals and insurers, they bear no clear relationship to actual resource usage during treatment. It is therefore quite probable that the US price differential between early- and late-stage cancer treatment arises as much from a negotiated agreement between care suppliers and purchasers as it does from any differential use of inputs.

By far the largest cost component over the 3 year period is the main treatment episode, 65 and 69% in the study and control groups, respectively. Although polypectomy offers the principal hope for economies in treatment costs resulting from screening, in only 15 cases did simple polypectomy represent the sole treatment episode (ten screen-detected, one no-response, four controls). All of these cases were stage A cancers, with a mean treatment cost of £257 (s.d. 142). However, the fact that a cancer is at a very early stage or confined within a polyp does not *guarantee* that treatment can be effected successfully by polypectomy alone (Langer *et al.*, 1984; Morson *et al.*, 1984; Russell *et al.*, 1990), nor does it preclude the possibility of recurrence at a later stage (Lotfi *et al.*, 1986). Indeed, the remaining stage A cancers in the combined sample (80%) all required resections at a higher cost, although at a lower cost than that necessitated by the treatment of stages B and C cancers. In six cases, re-admission occurred, after-care was required in nine cases, whilst seven patients experienced both re-admission and after-care.

It is evident that any treatment cost advantage which might have been anticipated as a result of screening has been substantially eroded by the high costs incurred by the no-response group. The proportion of costs per case in excess of £5,000 is considerably higher for this group than for both control and screen-detected cases (21.0%, compared with 11.2 and 10.4% respectively). The reasons for this cost difference are, at present, unclear. From the results, it is possible that cancers in no-response cases are more difficult to treat or that no-response patients are worse affected by both disease and treatment.

### Conclusions

Accepting the Nottingham finding, it accordingly follows that substantial economies in treatment costs during the 3 years following initial diagnosis should not be anticipated following the implementation of a colorectal cancer screening programme. This is because detection at early-, as opposed to late-, stage appears to make no significant difference to hospital treatment costs. Indeed, if one were to argue that a screening programme would detect a cancer at the asymptomatic early stage some years in advance of detecting it symptomatically at the late stage, then the *discounted* treatment cost would actually be higher under the screening scenario. Sizeable treatment cost economies as a result of screening would only become evident if the staging distribution were to be 'rolled forward' such that the proportion of specifically stage A cancers came to dominate the total. Such a situation could only be envisaged under the assumptions that the programme were to be screening for incident cancers only, and had high sensitivity and compliance rates.

This result, however, should not be taken to pre-judge the cost-effectiveness, or otherwise, of such a programme. From the evidence presented, it is reasonable to conclude that the principal explanation of low treatment cost in the screen-detected cases is cheap and successful initial intervention. By contrast, the explanation for low cost in other categories is early patient death, obviating the need for treatment. There are already evidence from other sources that expected survival gains are strongly correlated with cancer stage at diagnosis (Jatzko *et al.*, 1992), and it is expected that the Nottingham trial will also demonstrate such gains when its survival results are eventually published. The implication is that, whilst treatment costs may presently show no differences under a screening *vs* a non-screening scenario, outcome benefits are likely to be superior under the former, given the difference in the staging distribution. Although important, treatments costs are only one element in the cost-effectiveness equation which remains to be fully identified.

The research was conducted within the Nottingham colorectal cancer screening trial, supported principally by the Medical Research Council.

### References

ALLISON, J.E. & FELDMAN, R. (1985). Cost benefits of Hemoccult screening for colorectal cancer. *Dig. Dis. Sci.*, **30**, 860–865.

BARRY, M.J., MULLEY, A.G. & RICHTER, J.M. (1987). The effect of workup strategy on the cost-effectiveness of fecal occult blood screening for colorectal cancer. *Gastroenterology*, **93**, 301–310.

EDDY, D.M., NUGENT, F.W., EDDY, J.F., COLLER, J., GILBERTSEN, V., GOTTLIEB, L.S., RICE, R., SHERLOCK, P. & WINAWER, S. (1987). Screening for colorectal cancer in a high-risk population. *Gastroenterology*, **92**, 682–692.

FINKLER, S.A. (1982). The distinction between costs and charges. *Ann. Inter. Med.*, **96**, 102–109.

HARDCASTLE, J.D., FARRANDS, P.A., BALFOUR, T.W., CHAMBERLAIN, J.O., AMAR, S.S. & SHELDON, M.J. (1983). Controlled trial of faecal occult blood testing in the detection of colorectal cancer. *Lancet*, **ii**, 1–4.

HARDCASTLE, J.D., THOMAS, W.M., CHAMBERLAIN, J., SHEFFIELD, J., BALFOUR, T.W., ARMITAGE, N.C., PYE, G., JAMES, P.D., AMAR, S.S. & MOSS, S. (1989). Randomised controlled trial of faecal occult blood screening for colorectal cancer: results for the first 107,344 patients. *Lancet*, **i**, 1160–1164.

HEINE, J.A. & ROTHENBERGER, D.A. (1991). Cost-effective management of colon and rectal cancer. *World J. Surg.*, **15**, 597–604.

JATZKO, G., LISBORG, P. & WETTE, V. (1992). Improving survival rates for patients with colorectal cancer. *Br. J. Surg.*, **79**, 588–591.

LANGER, J.C., COHEN, Z., TAYLOR, B.R., STAFFORD, S., JEEJEEBHOY, K.N. & CULLEN, J.B. (1984). Management of patients with polyps containing malignancy removed by colonoscopic polypectomy. *Dis. Colon & Rectum*, **27**, 6–9.

LOTFI, A.M., SPENCER, R.J., ILSTRUP, D.M. & MELTON, L.J. (1986). Colorectal polyps and the risk of subsequent carcinomas. *Mayo Clin. Proc.*, **61**, 337–343.

MILLER, A.B., CHAMBERLAIN, J., DAY, N.E., HAKAMA, M. & PROROK, P.C. (1991). (ed.). *Cancer Screening*, Cambridge: Cambridge University Press.

MORSON, B.C., WHITEWAY, J.E., JONES, E.A., MACRAE, F.A. & WILLIAMS, C.B. (1984). Histopathology and prognosis of malignant colorectal polyps treated by endoscopic polypectomy. *Gut*, **25**, 437–444.

NEUGUT, A.I. & FORDE, K.A. (1988). Screening colonoscopy: has the time come? *Amer. J. Gastroenterol.*, **83**, 295–297.

POLLARD, S.G., MACFARLANCE, R. & EVERETT, W.G. (1989). Surgery for recurrent colorectal cancer – is it worthwhile? *Ann. Roy. Coll. Surgeons England*, **71**, 293–298.

RUSSELL, J.B., CHU, D.Z.J., RUSSELL, P., CHAN, C.H., THOMPSON, C. & SCHAEFER, R.F. (1990). When is polypectomy sufficient treatment for colorectal cancer? *Amer. J. Surg.*, **160**, 665–668.

SHIELL, A. (1991). *Economics and Intensive Care: from General Principles to Practical Implications.* Discussion Paper No. 80. Centre for Health Economics, University of York.

WAGNER, J.L., DUFFY, B., WADHWA, S. & JAKUBOWSKI, L. (1990). *Costs and Effectiveness of Colorectal Cancer Screening in the Elderly* (Background Paper No. 5). Office of Technology Assessment, Congress of the United States.

WALKER, A.R., WHYNES, D.K., CHAMBERLAIN, J.O. & HARDCASTLE, J.D. (1991*a*). The costs of screening for colorectal cancer. *J. Epidemiol. Commun. Health*, **45**, 220–224.

WALKER, A.R., WHYNES, D.K., HARDCASTLE, J.D. & CHAMBERLAIN, J.O. (1991*b*). The hospital costs of diagnostic procedures for colorectal cancer. *J. Clin. Epidemiol.*, **44**, 907–914.

WHYNES, D.K., WALKER, A.R. & HARDCASTLE, J.D. (1992). Cost-effective screening strategies for colorectal cancer. *J. Public Health Med.*, **14**, 43–49.